# Oral contraceptive pill-induced esophagitis: a rare cause of pill esophagitis in a Rwandan woman

**DOI:** 10.1093/omcr/omaf001

**Published:** 2025-03-28

**Authors:** Gordon P Bensen, Matthew R Bryan, Esther Nishimwe, Bao Sean Nguyen, Kenechukwu Chudy-Onwugaje

**Affiliations:** Harvard Medical School, 25 Shattuck Street, Boston, MA 02115, United States; Harvard Medical School, 25 Shattuck Street, Boston, MA 02115, United States; Gisenyi District Hospital, Gisenyi, Rubavu District, 00000, Rwanda; Division of Digestive Diseases, UCLA Health, 200 Medical Plaza, Los Angeles, CA 90024, United States; Department of Medicine, Memorial Hospital Belleville, 4500 Memorial Drive, Belleville, IL 62226, United States; Center for Global Health, Perelman School of Medicine, 3620 Hamilton Walk, Philadelphia, PA 19104, United States

**Keywords:** Esophagus, pill esophagitis, Esophageal ulcer, oral contraceptive pill (OCP)

## Abstract

We describe a case of oral contraceptive pill (OCP)-induced esophagitis in a 43-year-old Rwandan woman. She presented with epigastric and retrosternal pain and reported daily use of a combined OCP containing ethinyl estradiol and levonorgestrel. Upper endoscopy revealed a solitary, clean-based esophageal ulcer. Histopathologic evaluation excluded infectious causes, and she improved with proton pump inhibitor therapy and modification of pill consumption habits. This is the first reported case of OCP-induced esophagitis on the African continent and the fifth case overall, and it highlights the importance of awareness of this rare gastrointestinal adverse effect of a commonly used class of drugs, particularly in an area of the world that is promoting the increased uptake of female contraception as a tool for family planning.

## Introduction

Pill or drug-induced esophagitis is an infrequent condition that occurs when certain medications cause direct toxic injury to the esophageal mucosa, potentially leading to serious complications such as ulceration, hemorrhage, strictures, and perforation in rare cases [1–3]. Oral contraceptive pills (OCPs) are an exceedingly rare cause of pill esophagitis, with only four previous cases reported in the literature [4–6]. In this report, we describe the presentation of a Rwandan woman with OCP-induced esophagitis and highlight the preventative measures that can be taken to reduce its likelihood of occurrence. To our knowledge, this is the first reported case of OCP-induced esophagitis on the African continent.

## Case report

**Figure 1:**
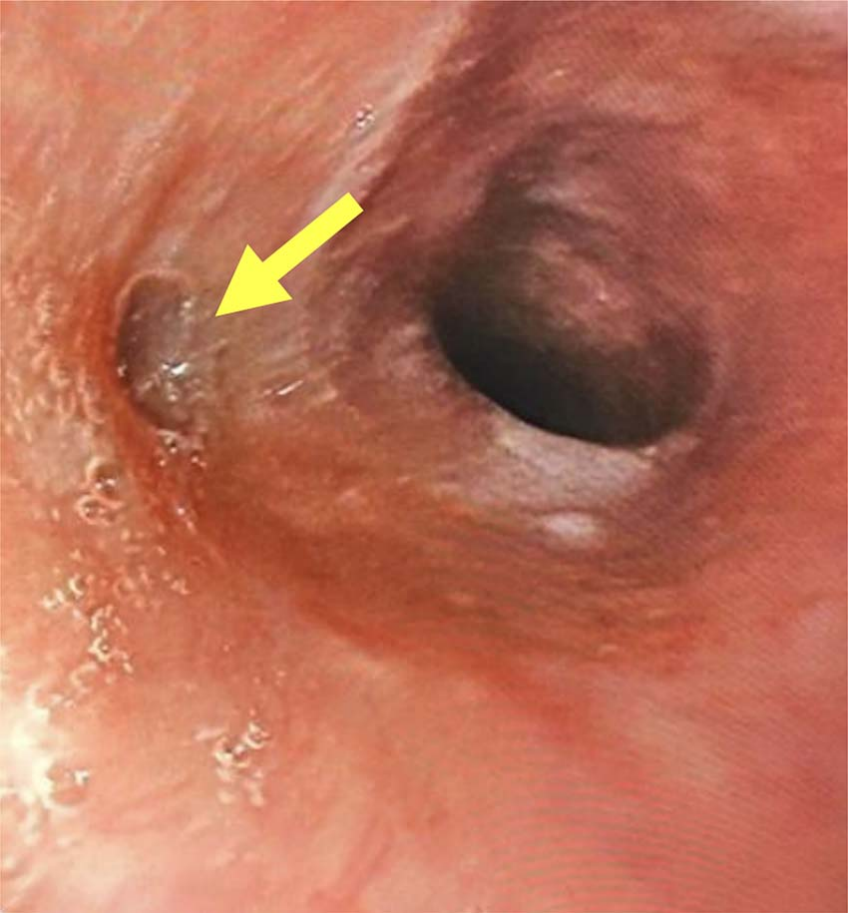
Solitary upper esophageal clean-based ulcer.

A 43-year-old Rwandan woman with no significant past medical history presented for evaluation of epigastric and retrosternal pain. She also reported anorexia and occasional vomiting but denied dysphagia or odynophagia. Besides daily use of a combined OCP containing ethinyl estradiol and levonorgestrel, she did not take any medications or consume alcohol. Physical examination was unremarkable except for slight elevation of blood pressure. Upper endoscopy performed by a visiting international medical team revealed a solitary, round, 1 cm clean-based esophageal ulcer, located 30 cm from the incisors (Fig. 1). The ulcer was biopsied, and treatment was initiated with twice daily omeprazole. Based on her history and endoscopic findings, a presumptive diagnosis of OCP-induced esophagitis was made, and the patient was counseled on lifestyle changes related to proper OCP consumption technique—consuming the pill with adequate fluids at least 30 min before laying supine and avoiding consumption of the pill before bedtime—to reduce the likelihood of pill esophagitis. Histopathologic evaluation of the biopsy specimen definitively ruled out infectious causes such as Herpes Simplex Virus or Cytomegalovirus. She had excellent clinical response with omeprazole and the recommended lifestyle changes. While unable to be performed due to limitations of the provider team’s physical location, evidence of mucosal healing on interval gastroscopy would have proven beneficial in ensuring therapeutic cure beyond subjective symptom resolution.

## Discussion

Pill esophagitis is a rare clinical diagnosis that typically results in injury to the esophageal mucosa through direct toxicity of the inciting drug. Proposed mechanisms of injury include alterations in local pH due to drug acidity, direct damage due to drug hyperosmolarity, and disruption of the normal barrier of the mucosa, all likely resulting from extended contact time between the offending agent and esophageal mucosa [3]. A great variety of drugs have been associated with esophageal injury, though pill esophagitis is most commonly associated with antibiotics (particularly doxycycline and other tetracyclines), non-steroidal anti-inflammatory drugs (ibuprofen, indomethacin, and aspirin), and bisphosphonates [3, 7]. While OCPs are widely prescribed around the globe, their tendency to cause pill esophagitis is not well known, with only four cases documented worldwide.

All previously reported cases involved healthy young women presenting with odynophagia and retrosternal pain in the context of OCP use. In 1991, Oren and Fich identified two cases of OCP-induced esophagitis: a 19-year-old female and a 20-year-old female, each taking levonorgestrel and ethinyl estradiol, were found to have three and one round esophageal ulcers, respectively, located 25–30 cm from the incisors. Both endorsed taking the pill without fluids either in the recumbent position or just prior to lying down [5]. In both cases, symptoms resolved in 4–5 days with proper ingestion technique and sucralfate therapy. Two additional cases have been reported, one in Germany in 1985 and the other in Turkey in 2005 [4, 6]. Similar to our observation in this Rwandan patient, these prior reports of OCP-induced esophagitis found that the ulcers were located between 25–31 cm from the incisors. It is well known that pill esophagitis is most likely to occur in the mid-esophagus, as it is the area with the greatest external compression from the aortic arch and left atrium [1, 3]. Our report adds to this limited body of evidence that identifies OCPs (levonorgestrel and ethinyl estradiol most commonly) as a cause of pill esophagitis.

OCP-induced esophagitis should be in the differential diagnosis for young female patients presenting with symptoms such as odynophagia, dysphagia, or burning retrosternal pain, and clinicians should consider a medication review in these instances. Though less common, vomiting and anorexia may also be present, as exemplified by our case. Awareness and inclusion of OCP-induced esophagitis in the differential diagnosis of esophageal symptoms in young women is particularly important for clinicians in low resource settings in Sub-Saharan Africa where access to diagnostic modalities like endoscopy is limited, but which have seen a concerted effort in recent years to increase uptake of female contraception as a tool for family planning [8, 9]. In all published cases of OCP-induced esophagitis, including the present case, subjects reported improper consumption of the pill. Clinicians, including general practitioners and family planning nurses, should not only be aware of this potential adverse event of OCPs but should also spend time to appropriately counsel patients on proper consumption technique. Protective practices include taking OCPs with a generous amount of water at least 30 minutes before bed and avoiding lying down immediately after ingestion. The goal is to reduce the amount of time the pill is in direct contact with the mucosa of the esophagus. Where OCP-induced esophagitis develops, proton pump inhibitors (PPIs), antacids, and sucralfate have been described to be effective for treatment [1, 10].

This case demonstrates the importance of thoroughly reviewing medications in young female patients who present with symptoms of esophagitis. Upper endoscopy with biopsy of esophageal ulcers is an important step in the evaluation of these patients, and OCP-induced esophagitis should be strongly considered after the exclusion of infectious, malignant, and autoimmune causes in the appropriate clinical context. Patient report of improper consumption of OCPs provides further supporting evidence for pill esophagitis as a clinical diagnosis and, in addition to medical therapy, counseling on proper pill consumption practices should be a vital component of patient care.
